# Circ‐Eif3c Carried by M2 Macrophage‐Derived Exosomes Mitigates Asthma Progression via miR‐15a‐5p/GSS/SOCS6 Axis Inhibition

**DOI:** 10.1155/mi/8350424

**Published:** 2026-03-02

**Authors:** Jiaying Yuan, Jiayi Zhao, Xiahui Ge, Xingxing Zhu, Li Li, Haoran Ni, Jian Fan, Yi Zhang, Yahong Sun, Yan Shang

**Affiliations:** ^1^ Department of General Practice, Shanghai Changhai Hospital, Naval Medical University (Second Military Medical University), Shanghai, 200433, China, chhospital.com.cn; ^2^ Department of Respiratory and Critical Care Medicine, Shanghai Changhai Hospital, Naval Medical University (Second Military Medical University), Shanghai, 200433, China, chhospital.com.cn; ^3^ Department of General Practice, Shanghai 411 Hospital, China RongTong Medical Healthcare Group Co., Ltd., Shanghai, 200081, China; ^4^ Department of Respiratory and Critical Care Medicine, Shanghai Ninth People’s Hospital, Shanghai Jiao Tong University School Of Medicine, Mohe Road, Shanghai, 201999, China, shsmu.edu.cn; ^5^ Department of Respiratory and Critical Care Medicine, Haining People’s Hospital, Haining, 314400, China

**Keywords:** asthma, circ-Eif3c, exosome, M2 macrophage, miR-15a-5p

## Abstract

**Background:**

In the study, we aimed to uncover potential therapeutic mechanisms concerning M2 macrophage‐derived exosomes in asthma.

**Methods:**

Exosomes were isolated from M0Φ‐Exos and M2Φ‐Exos. An ovalbumin (OVA)‐induced asthma mouse model or lipopolysaccharide (LPS)‐induced alveolar epithelial cells (AECs) were created to unravel the therapeutic mechanisms. High‐throughput sequencing was used to search for differentially expressed circRNA. Bioinformation analysis and luciferase report analysis were used to reveal the regulationship among circ‐Eif3c, miR‐15a‐5p, glutathione synthetase (GSS), and suppressor of cytokine signaling 6 (SOCS6).

**Results:**

The results showed that M2Φ‐Exos suppressed OVA‐induced inflammatory cytokine secretion and lung injury in mice. Next‐generation sequencing (NGS) showed that circ‐Eif3c was upregulated in M2Φ‐Exos. Circ‐Eif3c downregulation inhibited the therapeutic effect of M2Φ‐Exos. Bioinformation analysis confirmed that miR‐15a‐5p, GSS, and SOCS6 were the downstream targets of circ‐Eif3c, which were confirmed by luciferase report analysis. The overexpression of miR‐15a‐5p or the downregulation of GSS/SOCS6 reversed circ‐Eif3c’s protective effects on LPS‐induced AEC damage. The overexpression of circ‐Eif3c increased the therapeutic effect of M2Φ‐Exos.

**Conclusion:**

In conclusion, circ‐Eif3c‐enriched M2Φ‐Exos attenuated airway remodeling by restoring the function of AECs.

## 1. Introduction

Asthma affects more than 300 million people, and its incidence worldwide is increasing. It is a chronic respiratory trait that imposes high economic costs on individuals and healthcare systems over the long course of the illness [1]. Asthma is a heterogeneous trait in which genetic and environmental exposure are major pathogenic factors. Asthma is identified by airway inflammation, hypersecretion of mucus, airway hyperresponsiveness (AHR), variable airflow obstruction, and airway remodeling, which includes increased goblet cell counts, subepithelial fibrosis, epithelial damage, and lung blood vessel damaged [2, 3]. Despite significant advances in medical and basic investigations, asthma is a burden for both adolescents and adults and causes remarkable morbidity. Traditional treatments are efficient for people with mild‐to‐moderate asthma but are less effective for patients with severe asthma [4, 5]. Discovering new molecules that participate in asthma progression could provide more accurate asthma treatment targets.

Macrophage polarization profoundly affects asthma pathogenesis [6, 7]. On local microenvironment exposure, recruited macrophages might be polarized into a typically activated M1 (proinflammatory properties) or an activated M2 phenotype (anti‐inflammatory property). Macrophage polarization is highly relevant to asthma occurrence [8]. Regulatory processes of macrophage polarization include complex activities among different chemokines, cytokines, immunomodulatory cells, and transcription factors [9, 10].

Studies have found that macrophages regulate the microenvironment by exosomes. Exosomes are small extracellular vesicles (30–100 nm in diameter) that enable intercellular interaction through various molecules, including proteins, lipids, and nucleic acids such as microRNAs (miRNAs), mRNA, and circRNA. Exosomes are involved in cell functions and multiple disease pathologies, including asthma [11]. Previous studies have found that miR‐370 carried by M2 macrophage‐derived exosomes decreased asthma progression by inhibiting the MAPK/FGF1/STAT1 axis [12]. Exosomes from mmu_circ_0001359‐modified ADSCs weaken airway remodeling by promoting Forkhead box O1 (FoxO1) signaling‐mediated M2‐like macrophage activation [13]. However, whether circRNA regulates the microenvironment is still largely unclear. Long‐term remodeling or AHR data are still lacking. Therefore, this study aimed to uncover potential therapeutic mechanisms concerning M2 macrophage‐derived exosomes in asthma.

## 2. Methods

### 2.1. Ethics Statement

The ethics committee at Changhai Hospital, Second Military Medical University approved the investigation (20210909).

### 2.2. Animals

Male C57BL/6 mice at 6–8‐weeks‐old were purchased from Shanghai Slac Animal Laboratory (Shanghai, China). The mice were kept in a pathogen‐free conditioned animal care facility with controlled room temperatures (22°C) and photoperiods (12‐h light/12‐h dark cycle).

### 2.3. Macrophage Extraction and Culture

Briefly, RAW cells (RRID:CVCL_UL71) cultivated in high‐glucose Dulbecco’s Modified Medium (DMEM, 12100046; Gibco, Billings, MT, USA) supplemented with 10% FBS (Gibco, 10099141) and 20 ng/mL macrophage colony‐stimulating factor (M‐CSF, SRP3221; Sigma–Aldrich, St. Louis, MO, USA) were used for M0 induced, and 1% penicillin‐streptomycin (Gibco, SV30010) was added in the DMEM. The BMDMs were cultured in 20 ng/mL IL‐4 (Sigma–Aldrich, SRP3211) for M2 polarization. The M2 and M0 macrophages were harvested for flow cytometry analysis.

### 2.4. Exosome Identification and Extraction

After 3 days of M2 macrophage culture under 20 ng/mL IL‐4 condition with DMEM, the supernatants were collected. The samples were centrifuged at 1000 × g for 10 min to remove cell debris and apoptotic cells. The supernatants were then centrifuged at 10,000 × g and 4°C for 0.5 h to remove the shed microvesicles. The samples were resuspended in culture medium, which was ultracentrifuged at 100,000 × g and 4°C for 70 min. The samples were washed in phosphate buffer saline (PBS) with centrifugation at 100,000 × g for an additional 0.5 h. The precipitates containing the exosomes were resuspended in PBS. We utilized western blotting and transmission electron microscopy to analyze the exosomes. We determined size by dynamic light scattering with a Nanosizer. The size distribution was plotted with the particle radius (nm) on the *X*‐axis and the percentage on the *Y*‐axis.

For the signaling pathway study, the circ‐Eif3c overexpression vector or siRNA against circ‐Eif3c (Shanghai GenePharma Co., Ltd.), miR‐15a‐5p mimics (5′‐UAGCAGCACAUAAUGGUUUGUG‐3′) or inhibitor (Shanghai GenePharma Co., Ltd.), and siRNA against SOCS6 (Shanghai GenePharma Co., Ltd.) were constructed and transfected into macrophage 48 before exosome isolation. For cDNA synthesis, mRNA of SOCS6 was reverse transcribed utilizing the One‐Step SYBR PrimeScript RT‐PCR kit (#DRR014A, Takara Biotechnology, Mountain View, CA, USA) and then insert it into the cDNA3.1 vector.

### 2.5. CircRNA Expression Profile Analysis

Exosome samples from M0 or M2Φ were applied for circRNA expression analysis. The exosome samples were gained during surgery and were frozen immediately at −80°C for next‐step usage. A human circRNA chip (ArrayStar, Rockville, MD, USA) was utilized, which included 5639 probes that were particular for mouse circRNA splicing sites. Immediately after sample hybridization and cleaning, a pair of control and epileptic samples was analyzed on the circRNA chips. Exogenous RNA derived from the External RNA Controls Consortium was applied as controls. CircRNAs were enriched by digesting linear RNA with RNase R (Epicenter, Madison, WI, USA). The labeled RNAs were scanned using the G2505C scanner (Agilent Technologies, Santa Clara, CA, USA). KangChen Biotech (Shanghai, China) made the circRNA microarray procedure.

### 2.6. Establishing a Mouse Model With Asthma

Ovalbumin (OVA, Sigma–Aldrich, A5503) was used to induce asthma. First, 20 g complete Freund’s adjuvant was emulsified in 1 mg aluminum hydroxide (Sigma–Aldrich, 239186). This was administrated via intraperitoneal injection in a total volume of 0.2 mL to each mouse on days 0, 7, and 14 to sensitize the mice to OVA. On day 15, every mouse was exposed to 1% OVA aerosol for 1 h. The mice in the control group were maintained with normal saline. In addition, the mice subjected to M2Φ‐Exos (vein injection) treatment were treated with 20 μg M2Φ‐Exos for 3 continuous days from day 20. To obtain the BALF, the lungs were lavaged three times with ice‐cold PBS (0.5 mL) and withdrawn each time using a tracheal cannula (a total volume of 1.5 mL). The collected BALF was centrifuged at 1000 × g for 15 min at 4°C, and the supernatants were collected and frozen at −80°C for subsequent assays.

### 2.7. Alveolar Epithelial Cell Culture

Alveolar epithelial cells (AECs) purchased from ScienCell were cultured at 37°C with 5% CO_2_ in DMEM culture medium supplemented with 100 units/mL penicillin/streptomycin and 10% FBS. To simulate the asthma model, AECs were cultured with 1 µg/mL LPS.

### 2.8. Flow Cytometry

Flow cytometry was used to assay AEC apoptosis post FITC‐conjugated annexin V and propidium iodide (PI) staining. The cells were cleaned twice and then adjusted to 1 × 10^6^ cells/mL in cold D‐Hanks buffer. Then, 10 μL annexin V‐FITC and an equivalent volume of PI were added to 100 μL cell suspension, which was incubated for 15 min at room temperature in the dark. Prior to analyses, 400 µL binding buffer was added to every sample without cleaning. Each assay was performed in triplicate at least.

### 2.9. Tubule Formation Assay

In vitro neovascularization was measured in human fibrin matrices. After the treatments, serum‐starved AECs were seeded in endothelial basal medium in plates coated with Matrigel (10^5^ cells/well in 6‐well plates) (BD Biosciences, Franklin Lakes, NJ, USA) and incubated at 37°C for 12 h.

### 2.10. Quantitative Real‐Time PCR

Total RNA was obtained utilizing TRIzol (Invitrogen; Thermo Fisher Scientific, Waltham, MA, USA) and following protocols. RNA was reverse transcribed utilizing the One‐Step SYBR PrimeScript RT‐PCR kit (#DRR014A, Takara Biotechnology, Mountain View, CA, USA). An ABI PRISM 7500 real‐time PCR system (Applied Biosystems, Foster City, CA, USA) was used with the following conditions: 42°C for 5 min, 95°C for 10 s; 40 cycles of 95°C for 5 s, 72°C for 0.5 min, and 55°C for 0.5 min. Three independent experiments were done for every sample. The data were analyzed by computing 2^−ΔΔ^Ct values. U6 and glyceraldehyde phosphate dehydrogenase were used as internal controls: mmu_circ_0014154 forward, 5‐CTGTAAAACCACAGCTAG‐3, and reverse, 5‐CTGTCTTCTGTACCGAAG‐3; mmu_circ_0014052 forward, 5‐GGCTGTAGCCTGCAGTG‐3, and reverse, 5‐CTGCCTTGCCTTCTTC‐3; mmu_circ_0014023 forward, 5‐GACTGATCGGAGGTTTG‐3, and reverse, 5‐GACCATCCTGGGAGCCTG‐3; mmu_circ_0001655 forward, 5‐CCATAGAGGATGATGAC‐3, and reverse, 5‐CTTCCAGTCTATTGTAC‐3; mmu_circ_0001638 forward, 5‐GGCAGGACATGTTAGC‐3, and reverse, 5‐GACTTAACCCTTGGTC‐3; mmu_circ_0013946 forward, 5‐GTGACGACATCTCGGAC‐3, and reverse, 5‐CACCACATTCGTTTAC‐3; mmu_circ_0013934 forward, 5‐CTGTCCCAAGATGAGATAC‐3, and reverse, 5‐CAACTTCTCCTCTATAG‐3; mmu_circ_0013900forward, 5‐GAAGAGGAGCCATCTCAG‐3, and reverse, 5‐GTCGCTGCTCTTCATGGTG‐3; mmu_circ_0001628 forward, 5‐GTTCCACCACCAACTGC‐3, and reverse, 5‐CAGTTGCACCATAGTAC‐3; miR‐15a‐5p forward, 5′GGGTAGCAGCACATAATGGTTTGTG3′, and reverse, 5′CAGTGCGTGTCGTGGAGT3′; SOCS6 forward, 5‐CTCTCACCATTGCTACCTC‐3, and reverse, 5‐TGAGTCCACTGAAGTTCCT‐3; U6 forward, 5′GCTTCGGCAGCACATATACTAAAAT3′, and reverse, 5′CGCTTCACGAATTTGCGTGTCAT3′; GAPDH forward, 5′CTTTGGTATCGTGGAAGGACTC3′, and reverse, 5′GTAGAGGCAGGGATGATGTTCT3′.

### 2.11. ELISA

IL‐1β (ab197742), TNF‐α (ab208348), and IL‐6 (ab222503) expressions were determined using ELISA kits (Abcam, Cambridge Science Park, UK) according to the manufacturer’s protocols.

### 2.12. Immunofluorescence and Immunohistochemistry Assays

Lung tissue samples were fixed in 10% formalin solution, embedded in paraffin, and sectioned at 5 μm. The tissue sections were stained with Masson’s trichrome for histological validations. CD31 immunofluorescence staining was made to validate histopathological angiogenesis pathology. A terminal deoxynucleotidyl transferase dUTP nick end labeling (TUNEL) kit was applied to characterize apoptotic cells. ROS was detected via dihydroethidium staining. The tissue sections were examined using a fluorescence microscope (Nikon, Tokyo, Japan) or Axiophot light microscope (Zeiss, Oberkochen, Germany) and photographed using a digital camera.

### 2.13. Luciferase Reporter Assay

The 3′‐UTR target sequence was predicted for miR‐15a‐5p miRNA in the GSS/SOCS6 gene or circ‐Eif3c by employing the TargetScan online tool. The 3′‐UTR mutant and WT GSS/SOCS6 or circ‐Eif3c were made and cloned into a pMIR firefly luciferase expression vector (#kl‐zl‐1015‐01, KeLei Biological Technology Co., Ltd.). For luciferase assays, HEK293T cells at 70% confluence were cotransfected with 500 ng pMIR‐GSS/SOCS6‐WT/pMIR‐GSS/SOCS6‐Mut or pMIR‐circRNA‐WT/pMIR‐circRNA‐Mut and 50 nM miR‐15a‐5p NC/mimics (Shanghai GenePharma Co., Ltd.) by applying a Lipofectamine 2000 transfection kit (Thermo Fisher Scientific). The luciferase activity was monitored through a dual‐luciferase reporter system (Promega). Five independent assays were made.

## 3. Statistical Analyses

Data are shown as means ± standard deviation (SD). Statistical analyses were performed using GraphPad Prism (La Jolla, USA) to find significances among groups. *p*‐Values ≤ 0.05 were regarded as statistically significant. Two‐tailed Student’s *t*‐tests were used to determine significant differences between two groups, and one‐way ANOVA with post hoc Bonferroni tests were used to determine significant differences among three or more groups.

## 4. Results

### 4.1. M2Φ‐Exos Suppress OVA‐Induced Inflammatory Cytokine Secretion and Lung Injury in Mice

OVA was used to induce asthma‐like symptoms in mice. Then, exosomes from M2Φ were used to inhibit OVA‐induced lung injury (Figure 1A). The results showed that the inflammatory cytokines TNF‐α, IL‐6, and IL‐1β in BALF increased in OVA‐induced asthmatic mice. M2Φ‐Exos treatment suppressed OVA‐induced inflammatory cytokine secretion (Figure 1B–D). Immunohistochemical detection of cell apoptosis in mouse lung tissues showed that M2Φ‐Exos treatment suppressed OVA‐induced lung injury (Figure 1E,F). Fibrosis in mouse lung tissue detected via Masson’s trichrome staining showed that M2Φ‐Exos treatment inhibited OVA‐induced pulmonary fibrosis (Figure 1G,H). Immunofluorescence staining for CD31 showed that M2Φ‐Exos treatment restored OVA‐induced pulmonary vascular injury (Figure 1I,J). ROS detection showed that M2Φ‐Exos treatment inhibited OVA‐induced pulmonary ROS accumulation (Figure 1K,L).

Figure 1M2Φ‐Exos inhibit OVA‐induced lung injury and inflammatory cytokine secretion in mice. (A) The flow chart of the experiment. (B–D) The levels of TNF‐α, IL‐1β, and IL‐6 in BALF were measured by ELISA kits. The data are presented as the mean ± SD.  ^∗∗^ *p* < 0.01,  ^∗∗∗^ *p* < 0.001 vs. control group. ^###^ *p* < 0.001 vs. OVA group. *N* = 6. (E,F) Apoptosis of cells determined by TUNEL staining. The data are presented as the mean ± SD.  ^∗^ *p* < 0.05,  ^∗∗∗^ *p* < 0.001 vs. control group. ^##^ *p* < 0.01 vs. OVA group. Scale bar: 50 μm. *N* = 6. (G,H) Fibrosis in mouse lung tissues measured by Masson’s trichrome staining. The data are presented as the mean ± SD.  ^∗∗^ *p* < 0.01,  ^∗∗∗^ *p* < 0.001 vs. control group. ^##^ *p* < 0.01 vs. OVA group. Scale bar: 50 μm. *N* = 6. (I,J) CD31 expression in mouse lung tissues measured by immunofluorescence. The data are presented as the mean ± SD.  ^∗^ *p* < 0.05 vs. control group. ^###^ *p* < 0.001 vs. Scale bar: 50 μm. *N* = 6. (K,L) ROS levels in mouse lung tissues measured by dihydroethidium staining. The data are presented as the mean ± SD.  ^∗∗^ *p* < 0.01,  ^∗∗∗^ *p* < 0.001 vs. control group. ^###^ *p* < 0.001 vs. OVA group. Scale bar: 100 μm. *N* = 6.

**Figure:**
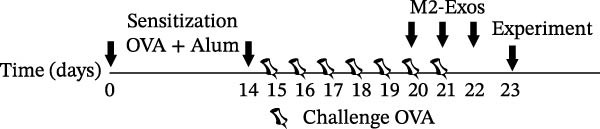
(A)

**Figure:**
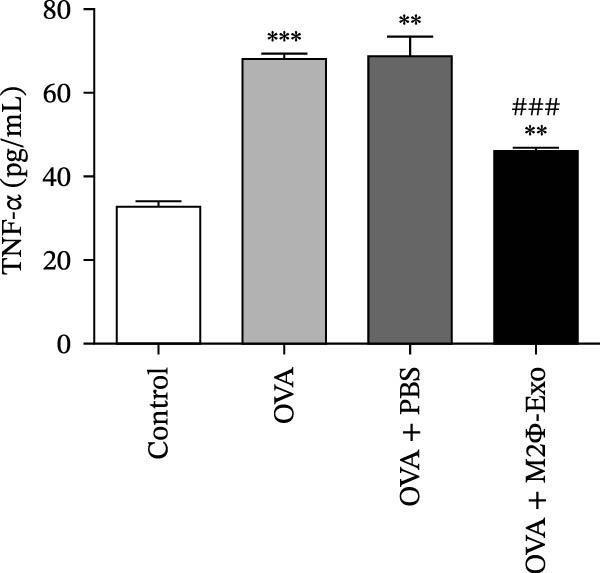
(B)

**Figure:**
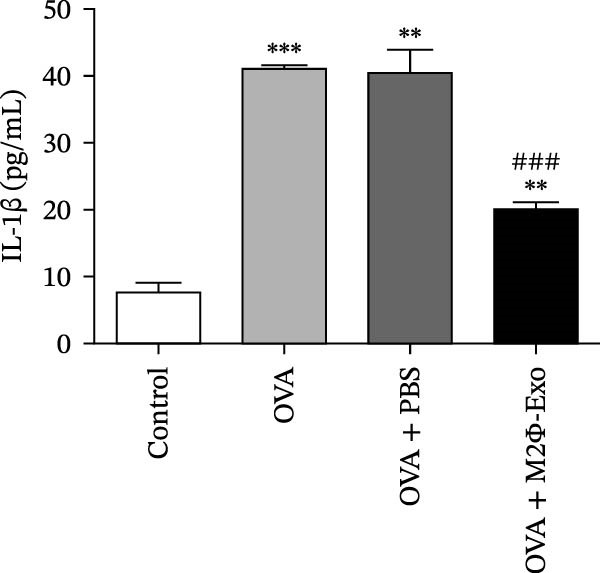
(C)

**Figure:**
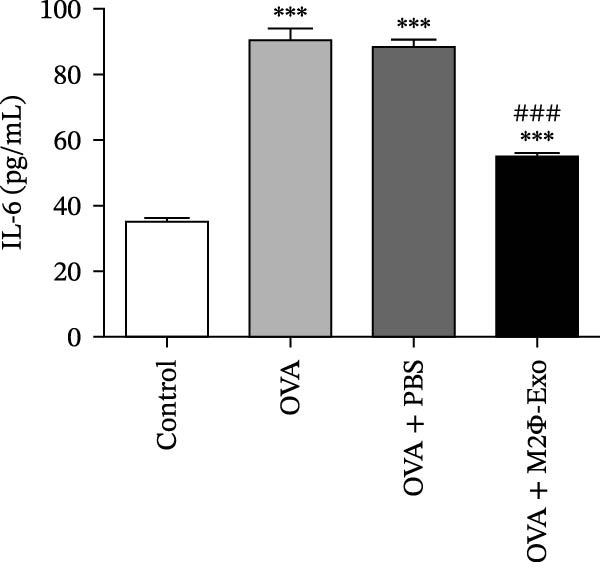
(D)

**Figure:**
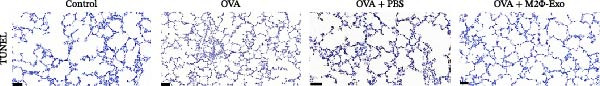
(E)

**Figure:**
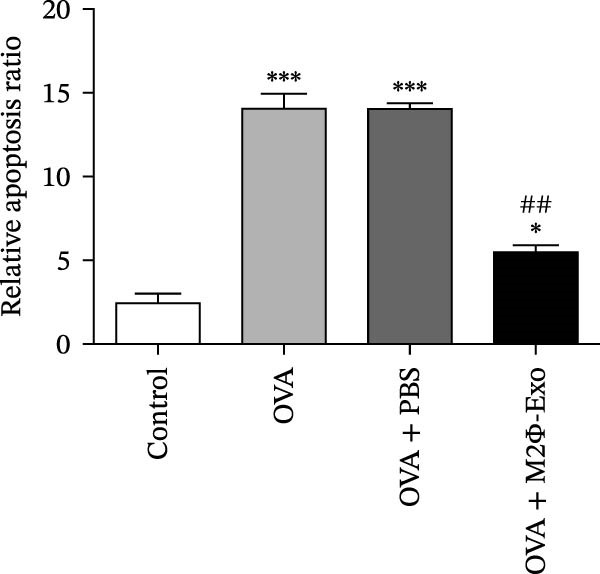
(F)

**Figure:**
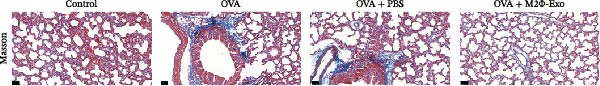
(G)

**Figure:**
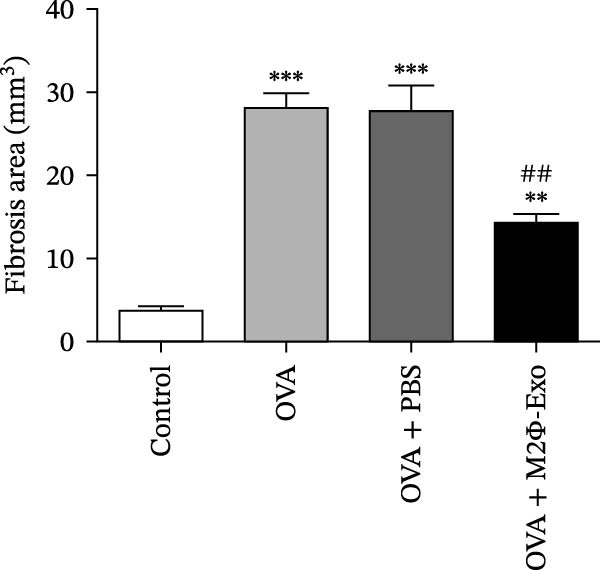
(H)

**Figure:**
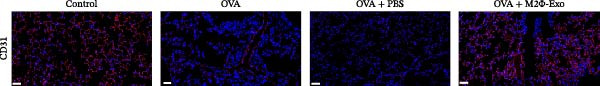
(I)

**Figure:**
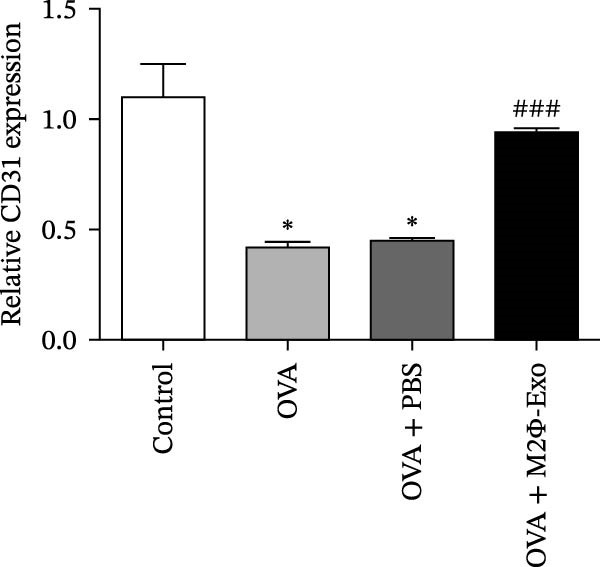
(J)

**Figure:**
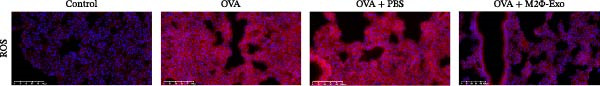
(K)

**Figure:**
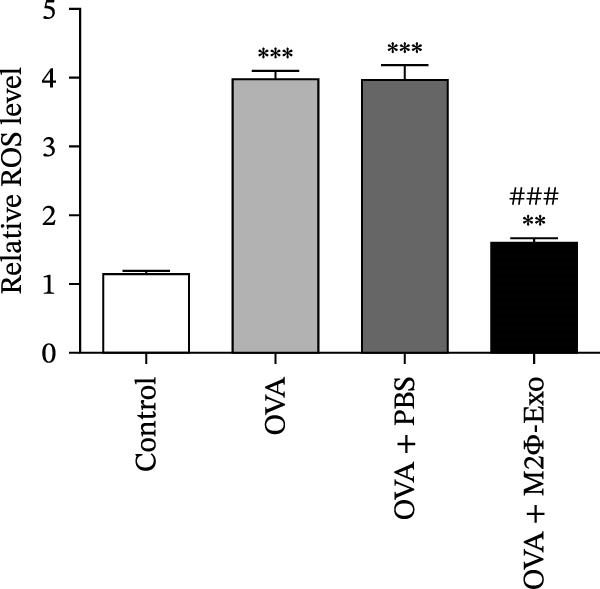
(L)

### 4.2. Circ‐Eif3c Plays an Important Role in M2 Macrophage‐Derived Exosomes

An accumulation study confirmed that circRNA function is important in microenvironment regulation [14]. To reveal whether circRNA plays a role in the M2Φ‐Exos‐mediated therapeutic effect on asthma, next‐generation sequencing (NGS) was utilized to detect different circRNA expressions between M0 and M2 macrophage‐derived exosomes (Figure 2A). RT‐qPCR was used to confirm the upregulation of circRNA mmu_circ_0001628, mmu_circ_0013900, mmu_circ_0013934, mmu_circ_0013946, mmu_circ_0001638, mmu_circ_0001655, mmu_circ_0014023, mmu_circ_0014052, and mmu_circ_0014154. Outputs showed that mmu_circ_0001628 expression was significantly increased (Figure 2B). Bioinformatics analysis (https://www.circbase.org/cgi-bin/simplesearch.cgi) confirmed the location of mmu_circ_0001628 on chromosome chr7:133695488‐133695852, which was constructed from the Eif3c gene and had a length of 364 bp. Therefore, mmu_circ_0001628 was also named circ‐Eif3c (Figure 2C).

Figure 2Circ‐Eif3c plays an important role in M2 macrophage‐derived exosomes. (A) NGS shows the expression of circRNA between M0 and M2 macrophage‐derived exosomes. (B) RT‐qPCR detection shows the expression of upregulated circRNA. *N* = 3. (C) Bioinformatics explores (https://www.circbase.org/) the chromosome location and origin of circ‐Eif3c.

**Figure:**
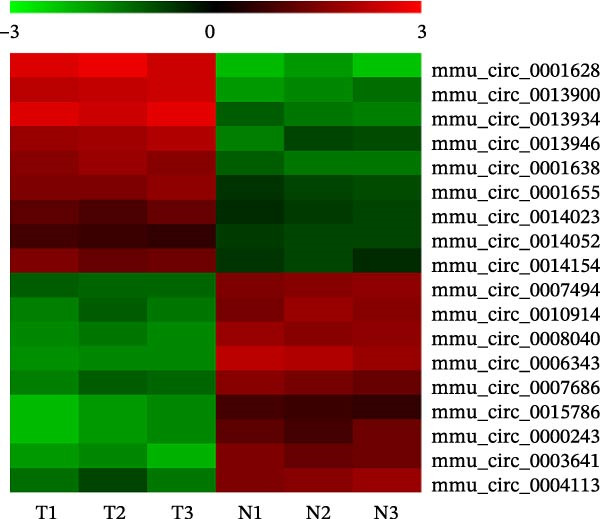
(A)

**Figure:**
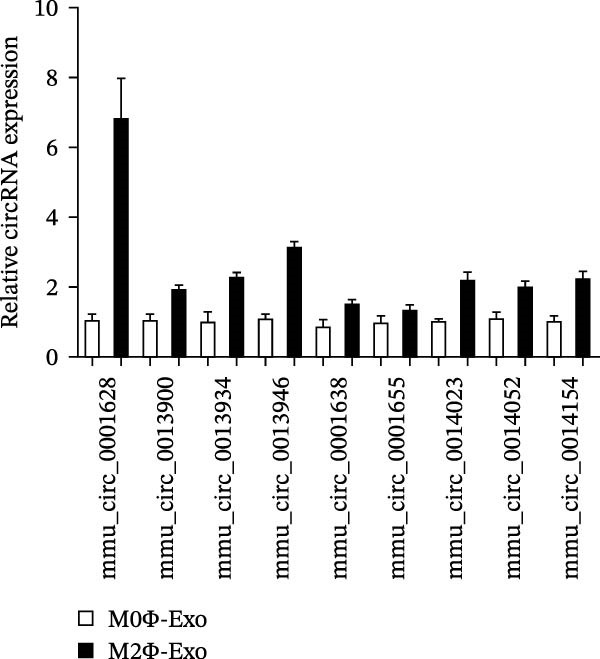
(B)

**Figure:**
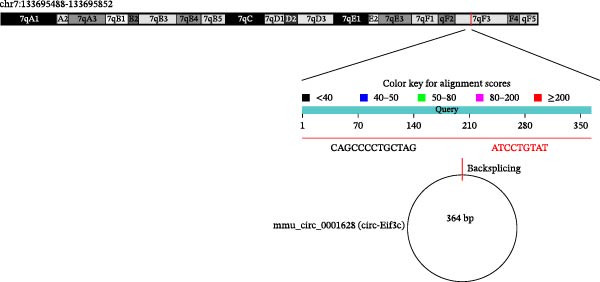
(C)

### 4.3. Downregulation of Circ‐Eif3c Inhibits the Therapeutic Effect of M2Φ‐Exos

To confirm whether circ‐Eif3c plays a role in the M2Φ‐Exos‐mediated therapeutic effect on asthma, siRNA against circ‐Eif3c was made and transfected into M2Φ. ELISAs for the inflammatory cytokines TNF‐α, IL‐1β, and IL‐6 in BALF showed that downregulation of circ‐Eif3c reduced the therapeutic effect of M2Φ‐Exos in OVA‐induced inflammatory cytokine secretion (Figure 3A–C). Immunohistochemical detection of cell apoptosis in mouse lung tissues showed that the downregulation of circ‐Eif3c decreased the therapeutic effect of M2Φ‐Exos in OVA‐induced lung injury (Figure 3D,E). Masson’s trichrome staining of mouse lung tissue showed fibrosis, and that the downregulation of circ‐Eif3c decreased the therapeutic effects of M2Φ‐Exos in OVA‐induced pulmonary fibrosis (Figure 3F,G). Immunofluorescence staining for CD31 showed that circ‐Eif3c downregulation decreased the therapeutic effect of M2Φ‐Exos in restoring OVA‐induced pulmonary vascular injury (Figure 3H,I). ROS detection showed that circ‐Eif3c downregulation decreased the therapeutic effect of M2Φ‐Exos in inhibiting OVA‐induced pulmonary ROS accumulation (Figure 3J,K).

Figure 3Downregulation of circ‐Eif3c inhibited the therapeutic effect of M2Φ‐Exos. (A–C) The levels of TNF‐α, IL‐1β, and IL‐6 in BALF were measured by ELISA kits. The data are presented as the mean ± SD.  ^∗∗∗^ *p* < 0.001 vs. M2Φ‐Exos. *N* = 6. (D,E) Apoptosis of cells determined by TUNEL staining. The data are presented as the mean ± SD.  ^∗∗∗^ *p* < 0.001 vs. M2Φ‐Exos. Scale bar: 50 μm. *N* = 6. (F,G) Fibrosis in mouse lung tissues measured by Masson’s trichrome staining. The data are presented as the mean ± SD.  ^∗∗∗^ *p* < 0.001 vs. M2Φ‐Exos. Scale bar: 50 μm. *N* = 6. (H,I) CD31 expression in mouse lung tissues measured by immunofluorescence. The data are presented as the mean ± SD.  ^∗∗∗^ *p* < 0.001 vs. M2Φ‐Exos. Scale bar: 50 μm. *N* = 6. (J,K) ROS levels in mouse lung tissues measured by dihydroethidium staining. The data are presented as the mean ± SD.  ^∗∗∗^ *p* < 0.001 vs. M2Φ‐Exos. Scale bar: 100 μm. *N* = 6.

**Figure:**
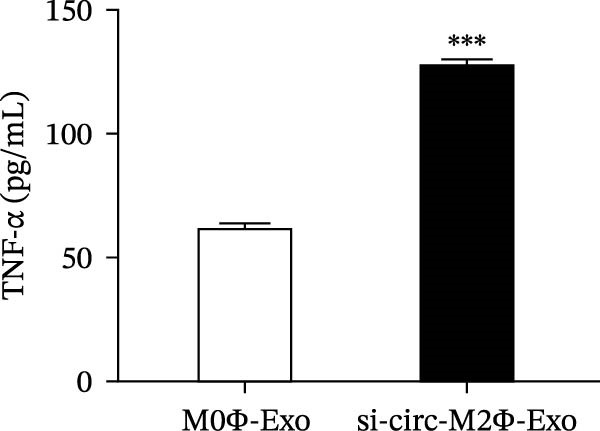
(A)

**Figure:**
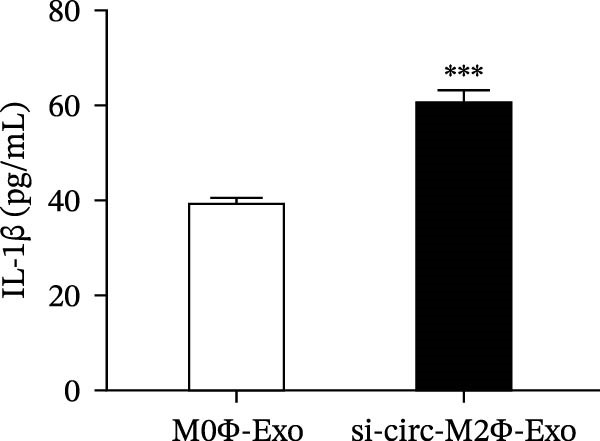
(B)

**Figure:**
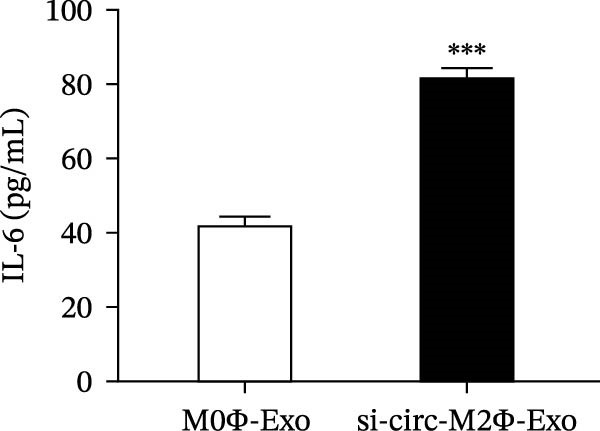
(C)

**Figure:**
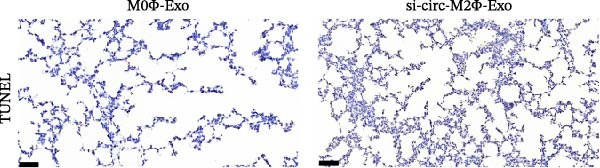
(D)

**Figure:**
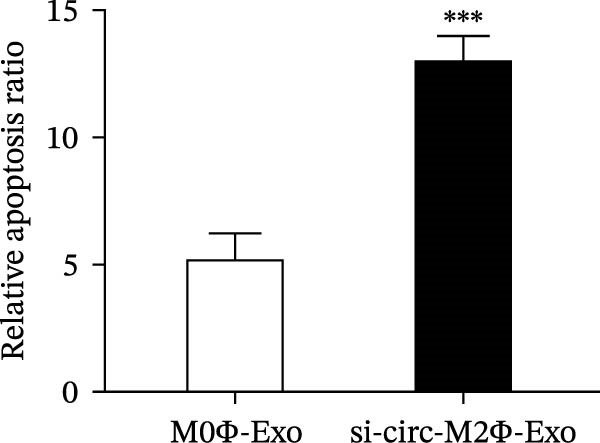
(E)

**Figure:**
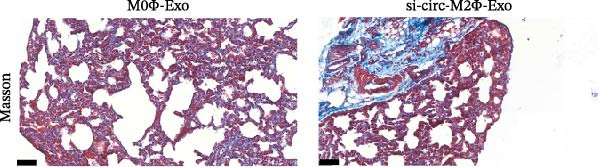
(F)

**Figure:**
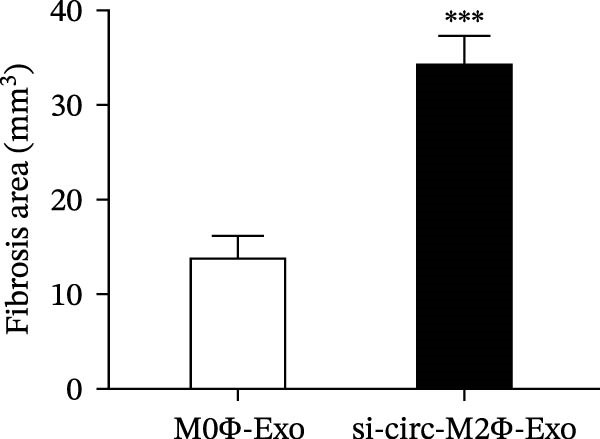
(G)

**Figure:**
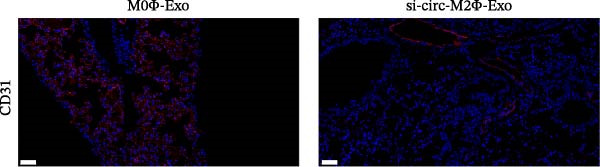
(H)

**Figure:**
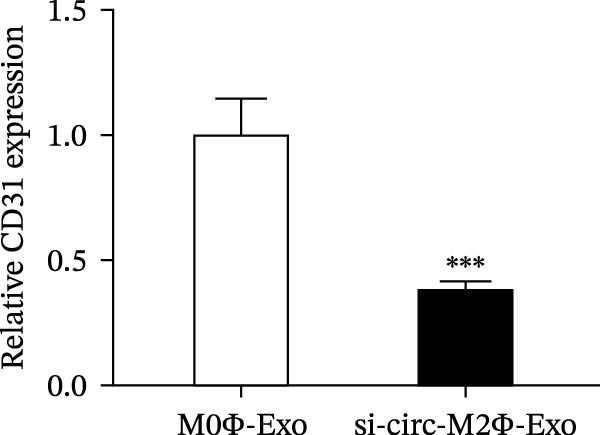
(I)

**Figure:**
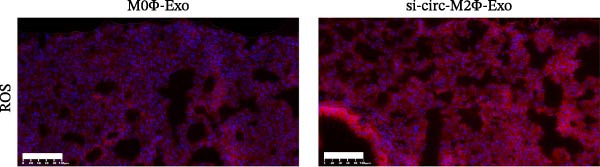
(J)

**Figure:**
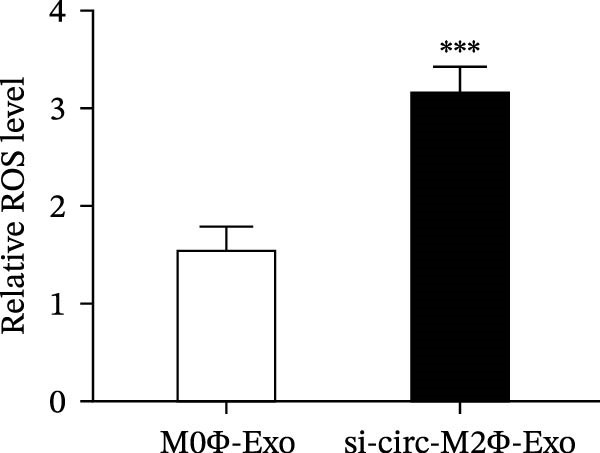
(K)

### 4.4. The miR‐15a‐5p, GSS, and SOCS6 Are Downstream Targets of Circ‐Eif3c

A predictive analysis was performed to identify the downstream targets of circ‐Eif3c. The analysis showed that miR‐15a‐5p was a circ‐Eif3c downstream target, and this was confirmed using a luciferase reporter assay (Figure 4A,B). Further study also confirmed that miR‐15a‐5p could interact with the 3’UTR of both GSS and SOCS6 (Figure 4C–F). RT‐qPCR showed that circ‐Eif3c expression in AECs was increased after transfected with circ‐Eif3c overexpression vector. And overexpression miR‐15a‐5p or downregulation GSS and SOCS6 cannot reverse the expression of circ‐Eif3c after transfection with circ‐Eif3c overexpression vector (Figure 4G). However, the upregulation of circ‐Eif3c downregulated miR‐15a‐5p and promoted GSS and SOCS6 expression. miR‐15a‐5p overexpression restored GSS and SOCS6 expression levels post‐circ‐Eif3c upregulation (Figure 4H–J).

Figure 4The miR‐15a‐5p, GSS, and SOCS6 are the downstream targets of circ‐Eif3c. (A–F) Luciferase reporter analysis shows the relationship between circ‐Eif3c and miR‐15a‐5p, miR‐15a‐5p and GSS, and miR‐15a‐5p and SOCS6. The data are presented as the mean ± SD.  ^∗∗∗^ *p* < 0.001. (G–J) RT‐qPCR detection shows the expression of miR‐15a‐5p, GSS, SOCS6, and circ‐Eif3c in airway epithelial cells (AECs) after transfection with circ‐Eif3c overexpression vector, miR‐15a‐5p mimic, siRNA against GSS (si‐GSS), and SOCS6 (si‐SOCS6). *N* = 3.

**Figure:**
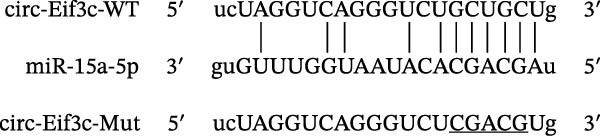
(A)

**Figure:**
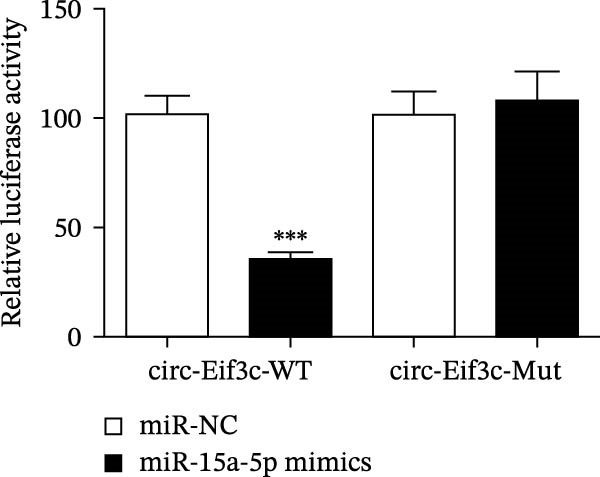
(B)

**Figure:**
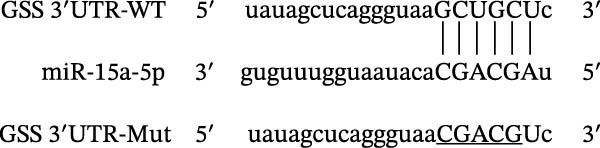
(C)

**Figure:**
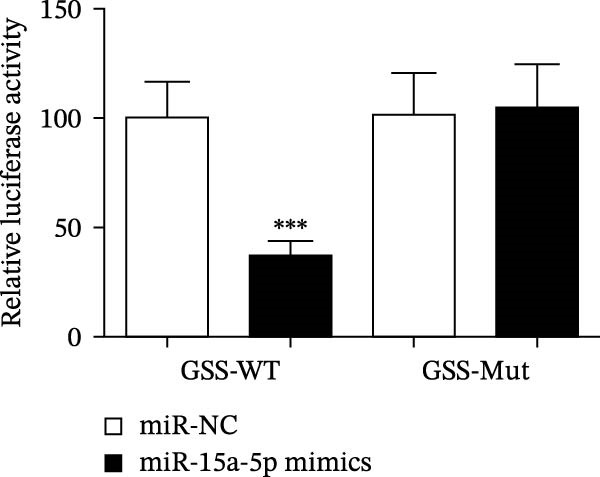
(D)

**Figure:**
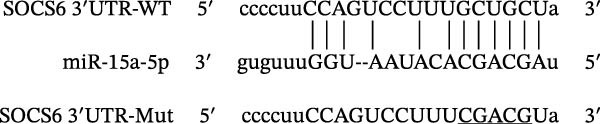
(E)

**Figure:**
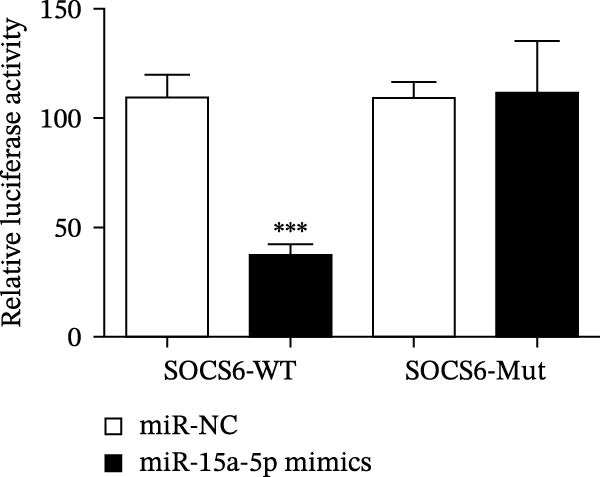
(F)

**Figure:**
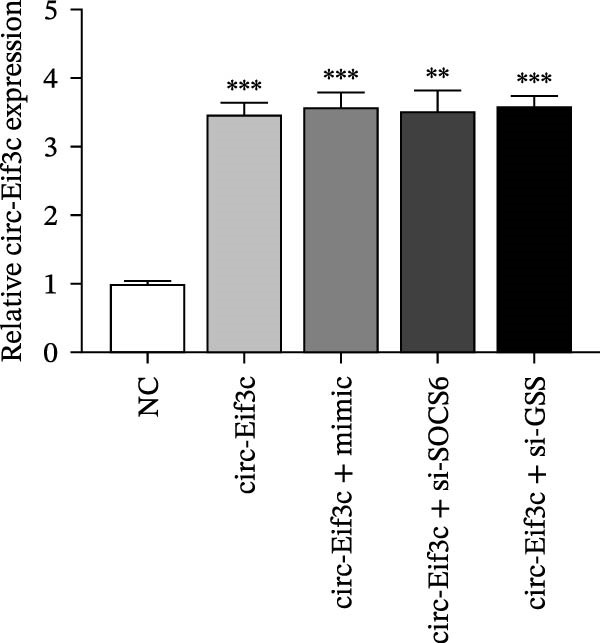
(G)

**Figure:**
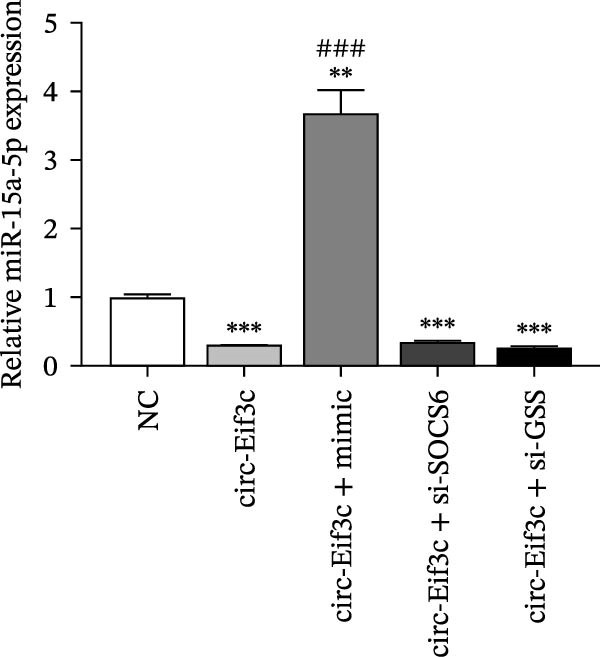
(H)

**Figure:**
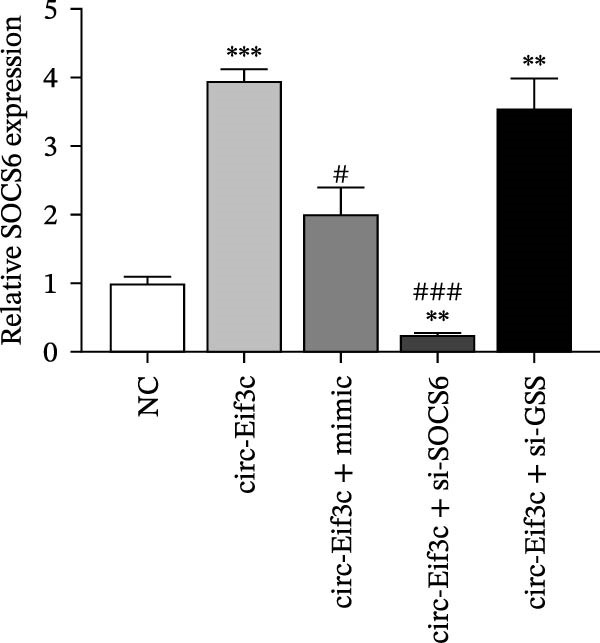
(I)

**Figure:**
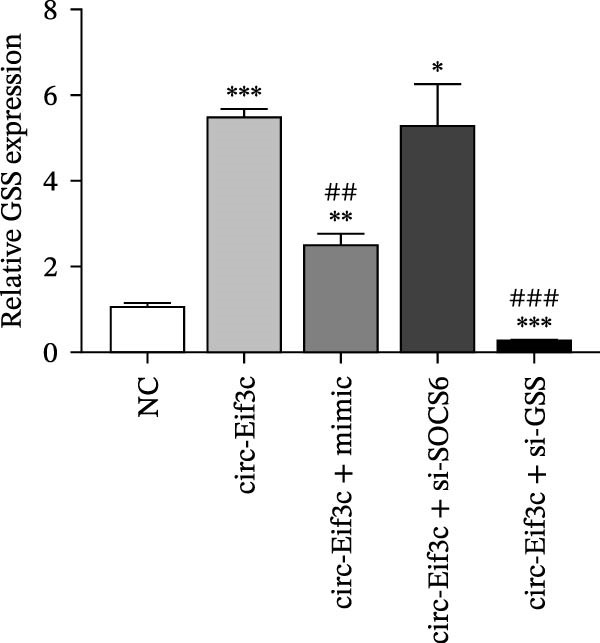
(J)

### 4.5. The miR‐15a‐5p Overexpression or GSS/SOCS6 Downregulation Reverses the Protective Effect of Circ‐Eif3c in LPS‐Induced AEC Damage

The analysis of apoptosis by flow cytometry showed that miR‐15a‐5p overexpression or GSS/SOCS6 downregulation reversed the protective effect of circ‐Eif3c in LPS‐induced AEC apoptosis (Figure 5A,B). ROS detection also confirmed that miR‐15a‐5p overexpression or GSS/SOCS6 downregulation reversed the protective effect of circ‐Eif3c in LPS‐induced AEC ROS accumulation (Figure 5C,D). Detecting the angiogenic differentiation ability showed that miR‐15a‐5p overexpression or GSS/SOCS6 downregulation reversed the protective effect regarding circ‐Eif3c to impair the LPS‐induced AEC angiogenic differentiation ability (Figure 5E,F).

Figure 5Overexpression of miR‐15a‐5p or the downregulation of GSS/SOCS6 reversed the protective effect of circ‐Eif3c in LPS‐induced AEC damage. (A,B) Apoptosis of AECs was assessed by flow cytometry using annexin V‐FITC staining, and the relative apoptosis ratio was calculated. Data are presented as the mean ± SD.  ^∗∗^ *p* < 0.01,  ^∗∗∗^ *p* < 0.001 vs. NC. ^###^ *p* < 0.001 vs. circ‐Eif3c. *N* = 3. (C,D) ROS levels in AECs were measured by dihydroethidium staining. Data are presented as the mean ± SD.  ^∗^ *p* < 0.05,  ^∗∗^ *p* < 0.01,  ^∗∗∗^ *p* < 0.001 vs. NC. ^###^ *p* < 0.001 vs. circ‐Eif3c. Scale bar: 50 μm. *N* = 3. (E,F) Angiogenic differentiation ability was detected. Data are presented as the mean ± SD.  ^∗∗^ *p* < 0.01,  ^∗∗∗^ *p* < 0.001 vs. NC. ^###^ *p* < 0.001 vs. circ‐Eif3c. Scale bar: 50 μm. *N* = 6.

**Figure:**
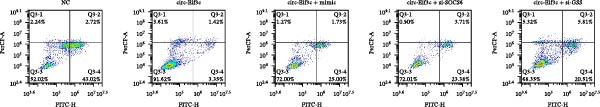
(A)

**Figure:**
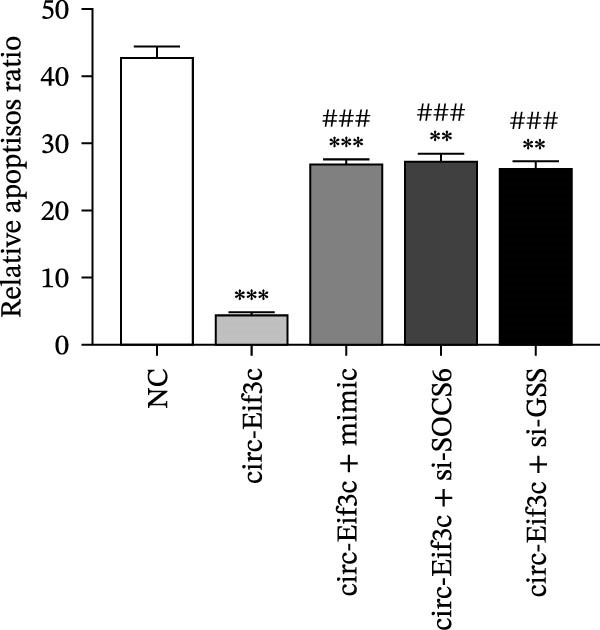
(B)

**Figure:**
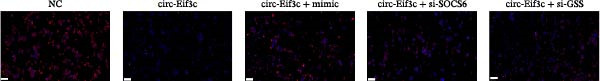
(C)

**Figure:**
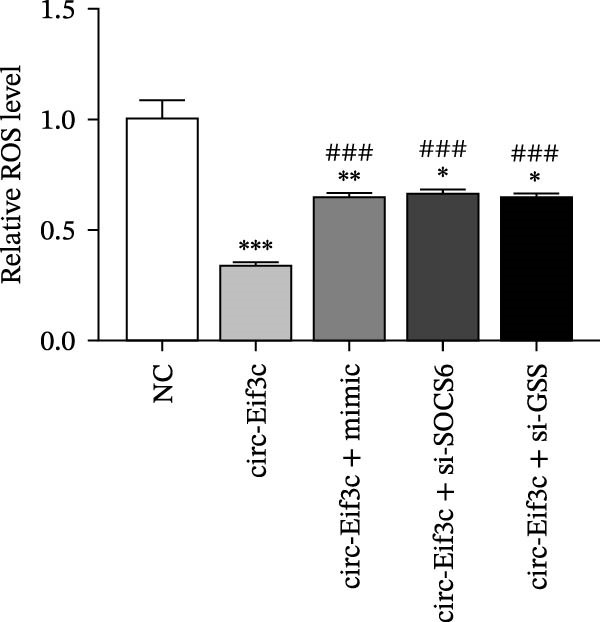
(D)

**Figure:**
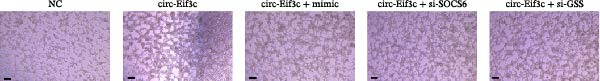
(E)

**Figure:**
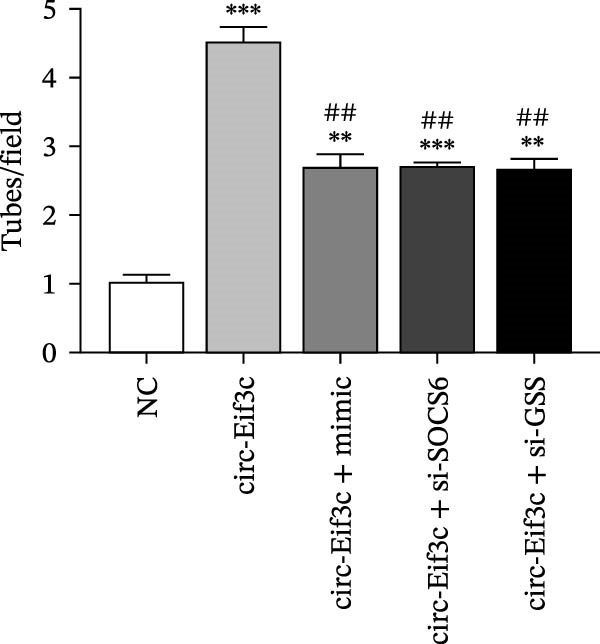
(F)

### 4.6. Circ‐Eif3c Overexpression Can Increase the M2Φ‐Exos Therapeutic Effect

The results showed that the overexpression of circ‐Eif3c increased the therapeutic effect of M2Φ‐Exos by suppressing OVA‐induced inflammatory cytokine secretion (Figure 6A–C). Immunohistochemical detection of cell apoptosis in mouse lung tissues showed circ‐Eif3c overexpression increased the therapeutic effect of M2Φ‐Exos by suppressing OVA‐induced lung injury (Figure 6D,E). The fibrosis detected in mouse lung tissue via Masson’s trichrome staining showed that circ‐Eif3c overexpression increased the therapeutic effects of M2Φ‐Exos by inhibiting OVA‐induced pulmonary fibrosis (Figure 6H,I). Immunofluorescence staining for CD31 showed that circ‐Eif3c overexpression increased the therapeutic effects of M2Φ‐Exos by restoring angiogenesis after OVA‐induced pulmonary vascular injury (Figure 6H,I). ROS detection showed that the overexpression of circ‐Eif3c increased the therapeutic effect of M2Φ‐Exos by inhibiting OVA‐induced pulmonary ROS accumulation (Figure 6J,K).

Figure 6Overexpression of circ‐Eif3c increased the therapeutic effect of M2Φ‐Exos. (A–C) The levels of TNF‐α, IL‐1β, and IL‐6 in BALF were measured by ELISA kits. The data are presented as the mean ± SD.  ^∗∗∗^ *p* < 0.001 vs. PBS. *N* = 6. (D,E) Apoptosis of cells determined by TUNEL staining. The data are presented as the mean ± SD.  ^∗∗∗^ *p* < 0.001 vs. PBS. Scale bar: 50 μm. *N* = 6. (F,G) Fibrosis in mouse lung tissues measured by Masson’s trichrome staining. The data are presented as the mean ± SD.  ^∗∗∗^ *p* < 0.001 vs. PBS. Scale bar: 50 μm. *N* = 6. (H,I) CD31 expression in mouse lung tissues was measured by immunofluorescence. The data are presented as the mean ± SD.  ^∗∗∗^ *p* < 0.001 vs. PBS. Scale bar: 50 μm. *N* = 6. (J,K) ROS levels in mouse lung tissues measured by dihydroethidium staining. The data are presented as the mean ± SD.  ^∗∗∗^ *p* < 0.001 vs. PBS. Scale bar: 100 μm. *N* = 6.

**Figure:**
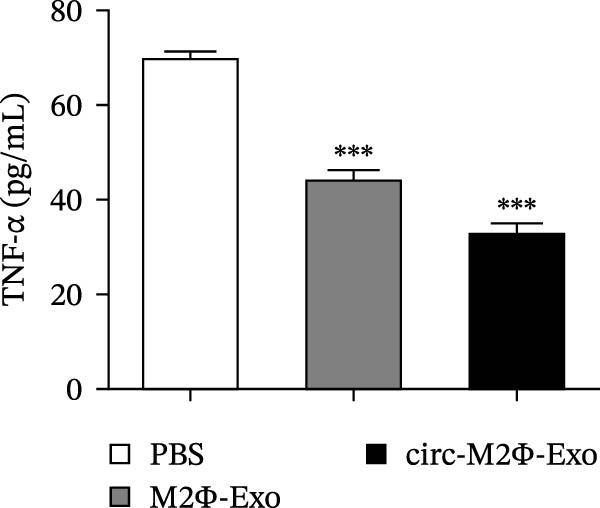
(A)

**Figure:**
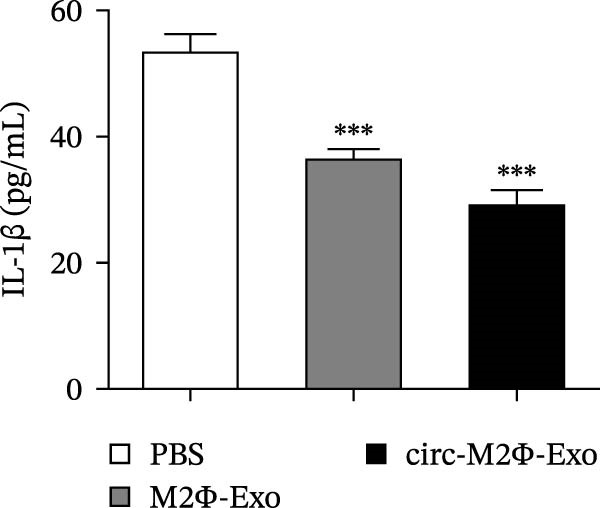
(B)

**Figure:**
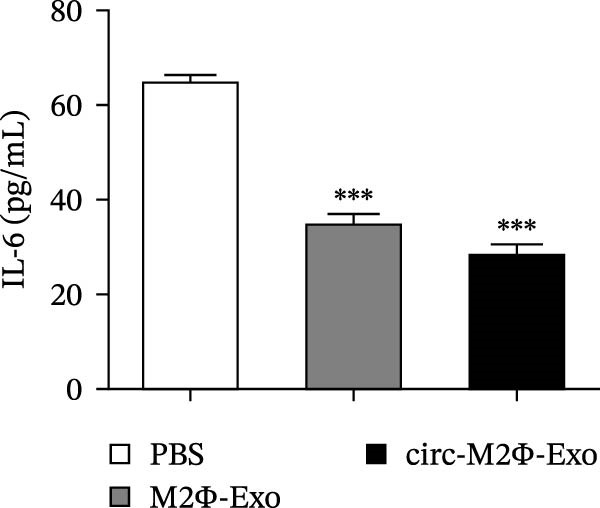
(C)

**Figure:**
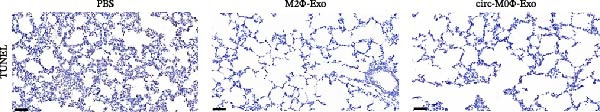
(D)

**Figure:**
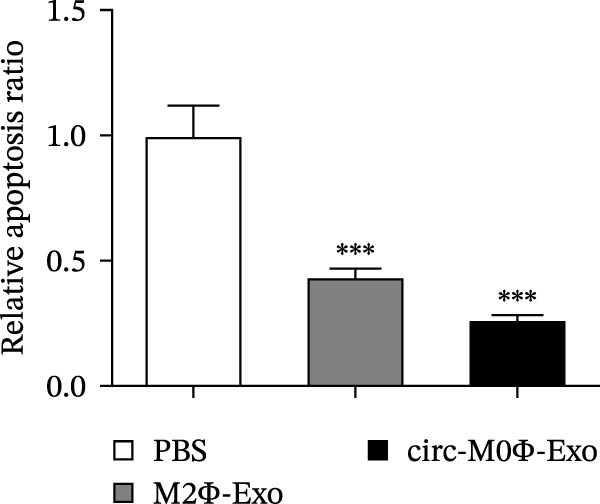
(E)

**Figure:**
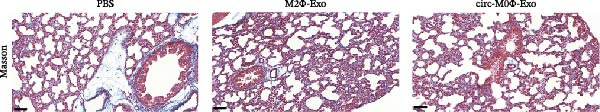
(F)

**Figure:**
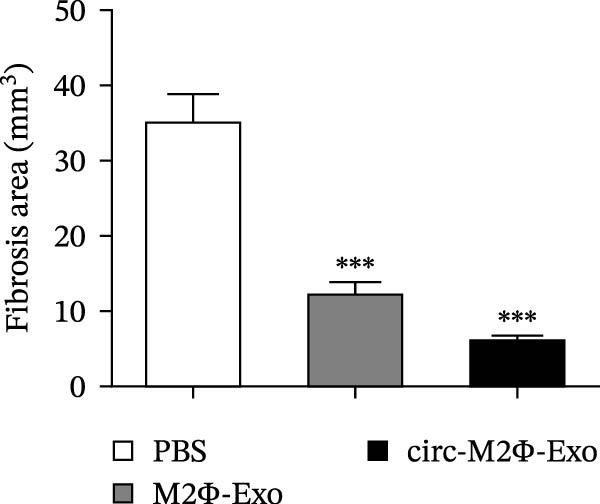
(G)

**Figure:**
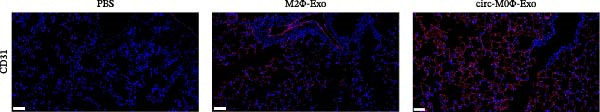
(H)

**Figure:**
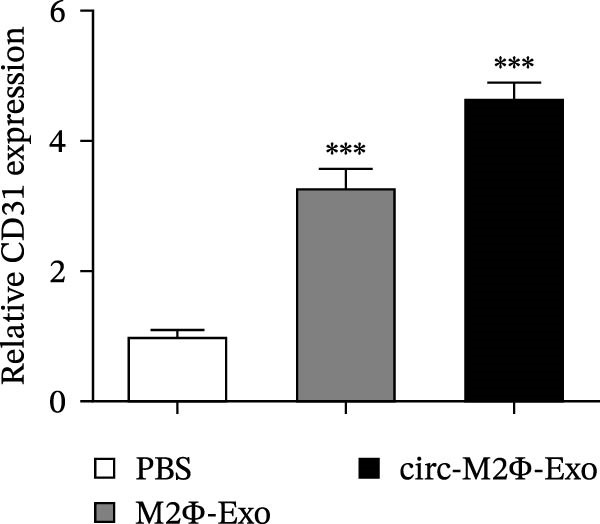
(I)

**Figure:**
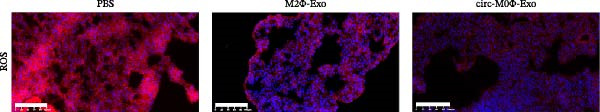
(J)

**Figure:**
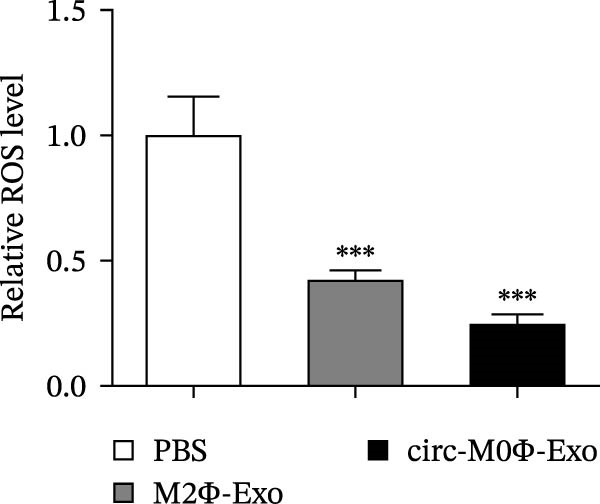
(K)

## 5. Discussion

Our previous research confirmed that FoxO1 signaling‐mediated M2‐like macrophage activation enhancements could attenuate airway remodeling [13]. In this study, we found that M2Φ‐Exos suppressed OVA‐induced inflammatory cytokine secretion and lung injury in mice. The accumulated investigations have confirmed that circRNA played an important role in airway microenvironment regulation [14]. Previous studies found that circ‐001372 reduced inflammation in OVA‐induced asthma via miR‐128‐3p and the Sirt1/NFAT5 signaling pathway [15]. Hsa_circ_0005519 increased IL‐6/IL‐13 via has‐let‐7a‐5p regulation in CD4^+^ T cells to affect asthma [16]. The present investigation found that circ‐Eif3c played an important function in M2 macrophage‐derived exosomes. Circ‐Eif3c downregulation suppressed the therapeutic effects of M2Φ‐Exos in OVA‐induced asthma.

Further studies confirmed that miR‐15a‐5p, GSS, and SOCS6 were the downstream targets of circ‐Eif3c. We evaluated the knockout of miR‐155 to alleviate airway inflammation and hyperresponsiveness in OVA‐sensitized mice [17]. In this study, circ‐Eif3c overexpression inhibited miR‐15a‐5p expression. miR‐15a‐5p overexpression reversed the protective effects of circ‐Eif3c in LPS‐induced AEC damage suggesting that circ‐Eif3c carried by M2 macrophage‐derived exosomes mitigated asthma progression by sponging miR‐15a‐5p.

The current investigation advised that miR‐15a‐5p could interact with the 3′UTR of GSS and SOCS6. GSS functions to maintain the REDOX balance, including ferroptosis. Ferroptosis causes the progression of various diseases, including asthma [18]. Inhibiting ferroptosis can alleviate asthma in vitro and in vivo [19, 20]. The current study also found that the overexpression of circ‐Eif3c promoted GSS expression, which inhibited asthma‐induced ROS accumulation, inflammatory factor expression, and ferroptosis. Overexpressing SOCS6 can reverse stress‐induced cell permeability and inflammatory response [21, 22]. In this study, we also found that the overexpression of circ‐Eif3c promoted SOCS6 expression, which promoted the functional recovery of AECs. Previous studies have confirmed that miR‐494‐3p intensified renal fibrosis, cell apoptosis, and EMT by targeting SOCS6 [23]. In this study, we found that the overexpression of circ‐Eif3c promoted SOCS6 expression, which inhibited asthma‐induced pulmonary fibrosis.

## 6. Conclusion

**Figure 7:**
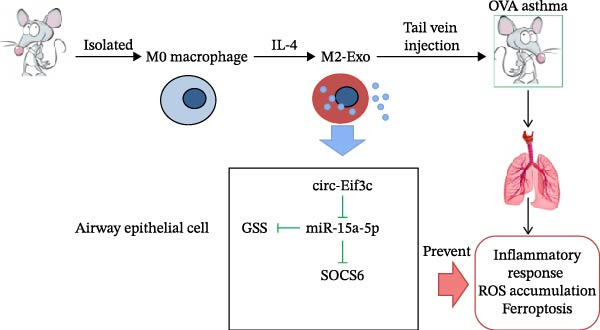
Mechanism diagram of circ‐Eif3c carried by M2 macrophage‐derived exosomes attenuated airway remodeling.

Taken together, circ‐Eif3c carried by M2 macrophage‐derived exosomes attenuated airway remodeling by restoring the function of AECs. Mechanism study has confirmed that circ‐Eif3c inhibit asthma‐induced pulmonary damage by regulation miR‐15a‐5p/GSS and miR‐15a‐5p/SOCS6 axis (Figure 7). The data presented circ‐Eif3c as a potential target for asthma therapy.

## Author Contributions

Jiaying Yuan, Jiayi Zhao, Xiahui Ge, Xingxing Zhu, and Yan Shang contributed to the study conception and design. All authors collected the data and performed the data analysis, contributed to the interpretation of the data and the completion of figures, and contributed to the drafting of the article.

## Funding

The study was supported by the National Key R&D Program of China (Grant 2024YFC3607500), the National Natural Science Foundation of China (Grant 82170033), Special Fund for “Research on Community Medicine and Health Management” in Shanghai (2024–40), Chang‐Feng Talent Fund of The First Affiliated Hospital of Naval Medical University (Second Military Medical University), Shanghai Oriental Talent Program Outstanding Project(Grant BJWS202428), the Zhejiang Health Science and Technology Project (Grant 2022KY394), and the Zhejiang Basic Public Welfare Research Project (Grant LQ22H010001).

## Disclosure

All authors contributed to the final approval of the submitted version.

## Ethics Statement

All applicable international, national, and/or institutional guidelines for the care and use of animals were followed. Ethical approval was given by the Ethics Committee of Shanghai Changhai Hospital, Naval Medical University (Second Military Medical University).

## Conflicts of Interest

The authors declare no conflicts of interest.

## Data Availability

The data that support the findings of this study are available from the corresponding author upon reasonable request.
